# Circadian Clock Regulates Epidermal Endocrine System in Homeostatic Skin Pigmentation

**DOI:** 10.1111/exd.70296

**Published:** 2026-06-10

**Authors:** Anya Zhu, Lingli Yang, Sylvia Lai, Fei Yang, Daisuke Tsuruta, Ichiro Katayama

**Affiliations:** ^1^ Department of Pigmentation Research and Therapeutics, Graduate School of Medicine Osaka Metropolitan University Osaka Japan; ^2^ College of Life Sciences Wuhan University Wuhan China; ^3^ Cosmetic Products Research, Research and Development Kao Corporation Odawara Japan; ^4^ Department of Dermatology, Graduate School of Medicine Osaka Metropolitan University Osaka Japan

**Keywords:** circadian rhythm, melanocytes, skin pigmentation, vitamin D metabolism, vitiligo

## Abstract

The circadian clock regulates multiple physiological processes in the skin, including local hormone synthesis and pigmentation. However, how circadian regulation interacts with epidermal endocrine signalling in melanocytes remains unclear. In this study, we investigated whether core circadian regulators influence melanogenesis in human melanocytes and explored their potential link to epidermal endocrine factors and pigmentary disease. Primary normal human epidermal melanocytes were analysed following siRNA‐mediated knockdown of the core clock genes basic helix–loop–helix ARNT‐like protein 1 (BMAL1) and CLOCK circadian regulator (CLOCK). Silencing either gene significantly reduced melanin synthesis and altered the expression of hormone‐related factors. Notably, expression of the vitamin D‐metabolizing enzyme CYP27A1 was markedly decreased after BMAL1 or CLOCK knockdown, changes that were associated with reduced melanogenic activity. Consistently, immunohistochemical analysis revealed diminished CYP27A1 expression in melanocytes within vitiligo lesions. These findings demonstrate that disruption of circadian clock regulators impairs melanogenesis and is accompanied by alterations in vitamin D‐related components. Our results suggest that circadian regulation may modulate pigmentation partly through effects on vitamin D metabolism, providing new insight into the mechanisms underlying pigmentary disorders.

## 1 Introduction

All living organisms exhibit circadian rhythms that regulate a wide range of physiological and behavioural processes [1]. At the cellular level, the circadian clock consists of transcriptional‐translational feedback loops that generate rhythmic gene expression [2]. The core regulatory loop involves BMAL1 and CLOCK, which form a heterodimer to activate the transcription of Period (PER1‐3) and Cryptochrome (CRY1‐2) genes. The translated PER and CRY proteins heterodimerize and translocate into the nucleus, where they inhibit their own transcription by interacting with the CLOCK:BMAL1 complex. In addition, the CLOCK:BMAL1 complex induces RORα and REV‐ERBα, which positively and negatively regulate BMAL1 transcription, respectively, forming an auxiliary loop that stabilizes circadian oscillation [3]. This intrinsic system enables cells to anticipate and adapt to daily environmental changes such as light, temperature, and nutrient availability.

Beyond the central pacemaker in the suprachiasmatic nucleus (SCN), peripheral circadian oscillators have been identified in various organs, including the skin, liver, and adrenal gland [4, 5]. In the skin, keratinocytes, melanocytes, and fibroblasts display autonomous circadian rhythmicity that regulates cellular proliferation, DNA repair, differentiation, and pigmentation [6, 7, 8, 9, 10, 11]. Several physiological processes of the skin, such as transepidermal water loss, barrier recovery, and melanin synthesis, exhibit diurnal variation, highlighting the importance of peripheral clocks in maintaining cutaneous homeostasis [12, 13, 14, 15, 16]. The rhythmic expression of clock genes in the epidermis not only reflects systemic cues but also responds directly to local stimuli such as ultraviolet (UV) exposure and temperature fluctuations, linking environmental light cycles to molecular processes in the skin [14, 17, 18, 19].

Accumulating evidence indicates that circadian disruption contributes to various skin disorders [15, 20, 21, 22]. Epidemiological studies have reported that night‐shift workers have an increased incidence of psoriasis [23]. Moreover, polymorphisms in BMAL1 have been identified in patients with non‐segmental vitiligo, suggesting that dysregulation of circadian genes may predispose individuals to pigmentary or autoimmune skin diseases [24].

In addition to its barrier and sensory roles, the skin also functions as a highly active local endocrine organ [25, 26, 27, 28]. It is capable of synthesizing, metabolizing, and responding to a variety of hormones and hormone‐like molecules, including vitamin D_3_, thyroid hormones, melatonin, cortisol, oestrogens, and light‐sensitive opsins. These endocrine factors, along with their biosynthetic enzymes and receptors, are expressed in multiple skin cell types, particularly keratinocytes and melanocytes. They modulate pigmentation, cellular proliferation, oxidative stress, and immune responses, thereby influencing both physiological adaptation and pathological processes [27, 29, 30, 31, 32]. Notably, many of these hormone systems are under circadian regulation in other tissues, suggesting that the cutaneous endocrine microenvironment may also be governed by local circadian control [25, 26, 27, 28, 29, 30]. However, the molecular interplay between core circadian regulators, melanocyte function, and local endocrine signalling remains poorly understood.

Melanocytes are the pigment‐producing cells in the epidermis. Recent studies have shown a specific peripheral clock characterized by rhythmic expression of core clock genes like BMAL1, PER1, and PER2, and that silencing these genes stimulates melanogenic activity, linking clock genes to melanin synthesis regulation [7, 32, 33]. Nevertheless, it remains unclear whether disruption of circadian clock genes affects the biosynthesis or receptor signalling of local hormones, such as vitamin D_3_, thyroid hormones, melatonin, cortisol, oestrogens, and opsins, in melanocytes.

In this study, we investigated how disruption of BMAL1 and CLOCK affects human melanocyte function and the biosynthesis and signalling of various local hormones, including vitamin D_3_, thyroid hormone, melatonin, cortisol, oestrogen, and opsins. This work aims to elucidate how circadian clock genes integrate endocrine pathways to regulate pigmentation and epidermal homeostasis.

## 2 Methods

### 2.1 Cell Lines and Cell Culture

**FIGURE 1:**
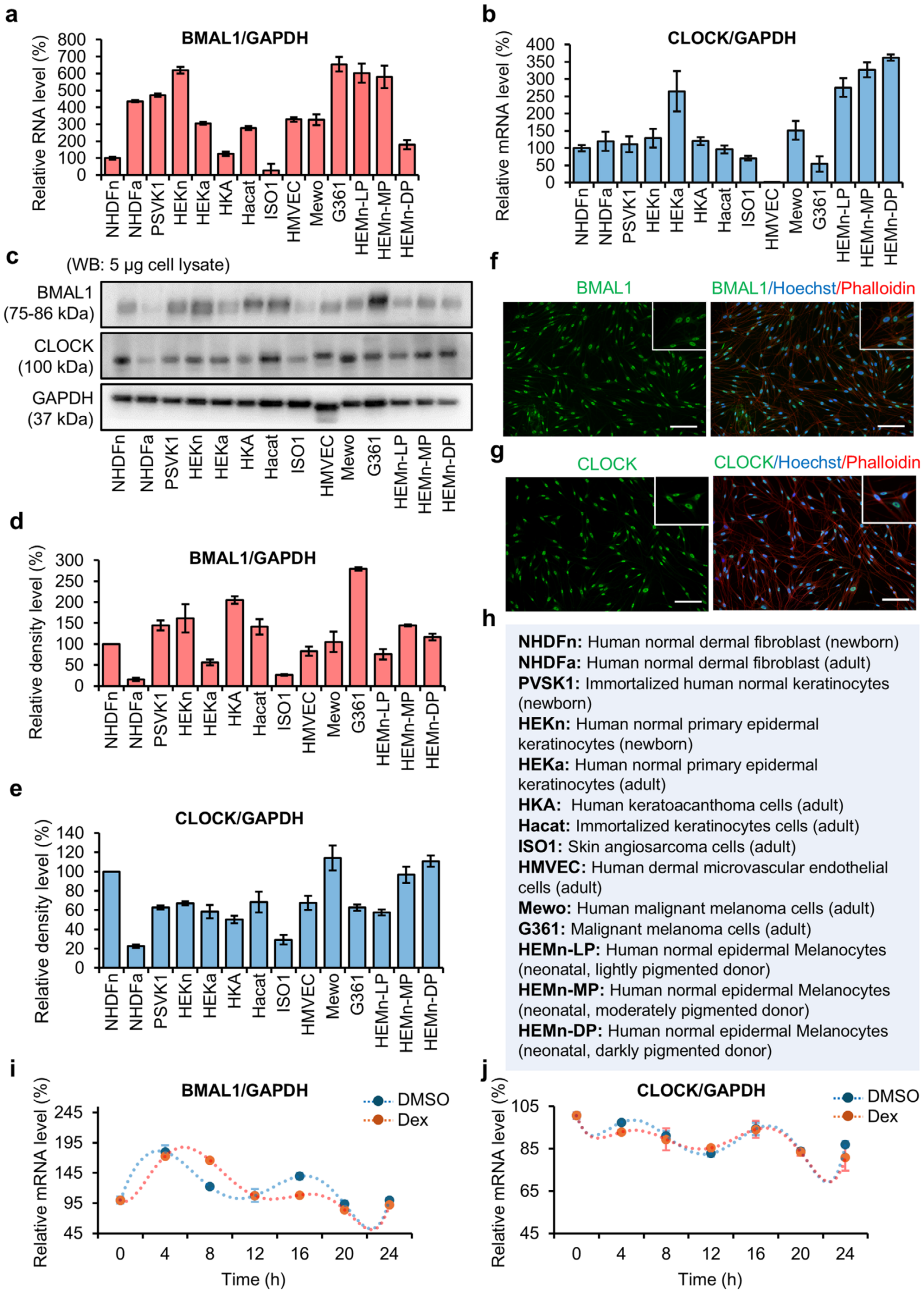
Expression of BMAL1 and CLOCK across diverse human skin cell types. Quantitative real‐time PCR analysis of BMAL1 (a) and CLOCK (b) mRNA expression in 14 human skin‐derived cell types. Transcript levels were normalized to GAPDH and presented relative to NHDFn. Representative Western blot analysis showing BMAL1 and CLOCK protein expression in whole‐cell lysates from the indicated cultured cells, with GAPDH serving as the loading control (c). Densitometric quantification of BMAL1 (d) and CLOCK (e) protein levels normalized to GAPDH. Immunofluorescence staining of cultured melanocytes (HEMn‐LP) showing the intracellular localization of BMAL1 (f) and CLOCK (g). BMAL1/CLOCK (green), Hoechst 33342 (blue), and Phalloidin (red). Insets highlight representative cells at higher magnification. Scale bar: 100 μm. Summary table listing detailed information on all cell lines used in this study, including species and tissue origin (h). Cultured human primary epidermal melanocytes (HEMn‐MPs) were synchronized with a 2‐h pulse of dexamethasone (DEX). BMAL1 (i) and CLOCK (j) mRNA expression levels were analysed by quantitative real‐time PCR at the indicated time points after synchronization (*t* = 0) with dexamethasone. Gene expression levels were normalized to GAPDH and expressed relative to the 0‐h control. DMSO was used as the vehicle control. Data in a, b, d, e, i, and j are shown as mean ± SD.

Human foreskin keratinocytes immortalized by infection with the pSV40 ori (PSVK1 cells) were purchased from the Japanese Collection of Research Bioresources (JCRB, Osaka, Japan) and cultured in KBM‐Gold Keratinocyte Basal Medium (Lonza, Walkersville, MD, USA) supplemented with the KGM‐Gold Bullet Kit (Lonza). HEKn (normal neonatal human epidermal keratinocytes) and HEKa (normal adult human epidermal keratinocytes) cells were obtained from Cascade Biologics (Portland, OR, USA) and cultured in Epilife medium supplemented with Human Keratinocyte Growth Supplement (HKGS) (GIBCO, Gaithersburg, MD, USA). The ISO‐HAS‐B (ISO1) skin angiosarcoma cell line was obtained from the Cell Resource Center for Biomedical Research of the Cell Bank (Sendai, Japan) and cultured in high‐glucose Dulbecco's modified Eagle's medium (DMEM) supplemented with 10% foetal bovine serum and 1% penicillin–streptomycin (Thermo Fisher). NHDF cells (human normal dermal fibroblasts) were acquired from Invitrogen (Thermo Fisher Scientific, Carlsbad, CA, USA) and cultured in low‐glucose DMEM supplemented with 10% foetal bovine serum and 1% penicillin–streptomycin (Thermo Fisher, Waltham, MA, USA). HKA (cell line derived from a keratoacanthoma of human skin), Mewo, and G361 (human melanoma cell lines) cells were procured from the Japanese Collection of Research Bioresources (JCRB, Osaka, Japan) and maintained in high‐glucose DMEM supplemented with 10% foetal bovine serum and 1% penicillin–streptomycin (Thermo Fisher, Waltham, MA, USA). HaCaT cells (human immortalized keratinocytes) were obtained from the American Type Culture Collection (ATCC, Manassas, VA, USA). HEMn‐MPs (normal human primary skin epidermal melanocytes from moderately pigmented neonatal foreskin) were obtained from Invitrogen (Thermo Fisher Scientific, Carlsbad, CA, USA) and cultured in Medium 254 (M‐254‐500; Thermo Fisher Scientific) supplemented with 1% (v/v) human melanocyte growth supplement (Thermo Fisher Scientific). HMVECs (human microvascular endothelial cells) were procured from Lonza (Walkersville, MD, USA) and cultured in endothelial basal medium‐2 (EBM‐2, Lonza). Detailed information about the sources of all cultured cells, including age and generation, is provided in Figure 1f. All cultures were maintained at 37°C in a CO_2_ incubator with an atmosphere of 5% CO_2_.

### 2.2 Human Skin Specimens

Paraffin‐embedded tissue sections of lesional skin from confirmed vitiligo patients in the progressive state (*n* = 4) and samples from corresponding sites of healthy donors (*n* = 4) were used in histo‐immunofluorescence staining. Written informed consent was obtained from all participants prior to study inclusion. The study was approved by the ethics committee of the Osaka Metropolitan University Faculty of Medicine (No. 4152).

### 2.3 Dexamethasone Synchronization of Circadian Rhythms

Cells were seeded in 6‐well plates and cultured to the appropriate density. For circadian synchronization, the culture medium was replaced with basal medium lacking growth supplements and containing 100 nM dexamethasone (Dex) (D4902‐100, Sigma‐Aldrich, MO, USA) prepared from a DMSO stock solution. Control cells received an equivalent volume of DMSO. Cells were incubated with Dex or DMSO for 2 h at 37°C. After treatment, cells were washed twice with pre‐warmed medium, and fresh complete medium containing growth supplements was added. The time point immediately after medium replacement was defined as 0 h. Cells were harvested at 0, 4, 8, 12, 16, 20, and 24 h after synchronization, and total RNA was extracted at each time point for quantitative real‐time PCR analysis.

### 2.4 RNA Interference

For siRNA‐mediated knockdown of BMAL1 and CLOCK, HEMn‐MPs were transfected with 30 nM pre‐designed Silencer Select siRNAs (si‐BMAL1: HSS100702; si‐CLOCK: HSS114292; si‐CYP27A1: 106239, Thermo Fisher Scientific) using Lipofectamine RNAi MAX (Invitrogen, Thermo Fisher Scientific) according to the manufacturer's instructions.

### 2.5 RNA Isolation and Real‐Time RT‐PCR Analysis

Total RNA was extracted from cell pellets using the Maxwell 16 LEV simplyRNA Tissue Kit (Promega, Madison, WI, USA) according to the manufacturer's instructions. RNA integrity was confirmed through gel electrophoresis. Subsequently, 100 ng of total RNA was reverse‐transcribed into first‐strand cDNA using the ReverTra Ace qPCR RT Master Mix (TOYOBO, Osaka, Japan).

Real‐time PCR was performed using THUNDERBIRD SYBR qPCR Mix (TOYOBO, Osaka, Japan; QPS‐201) on a QuantStudio 5 Real‐Time PCR System (Applied Biosystems, CA, USA). The primers used for real‐time PCR were as follows: human BMAL1 sense, 5′‐GCTTCTGCACAATCCACAGC‐3′ and antisense, 5′‐CACCCTGATTTCCCCGTTCA‐3′; human CLOCK sense, 5′‐CGAGCGCTCCCGAATTTTTA‐3′ and antisense, 5′‐AGGTATCTAGTGAGACTTGCCA‐3′; human short‐wave‐sensitive opsin 1 (OPN1SW) sense, 5′‐TATCTCTTCAGTGGGGCCGT‐3′ and antisense, 5′‐GGCTGCCGCAACTTTTTGTA‐3′; human opsin‐2 (OPN2) sense, 5′‐CATGACCATCCCAGCGTTCT‐3′ and antisense, 5′‐CTTGGACACGGTAGCAGAGG‐3′; human opsin‐3 (OPN3) sense, 5′‐CTACAAGTTCCAGCGGCTCC‐3′ and antisense, 5′‐CGAAGGTAAAGGTGACCCCG‐3′; human peropsin sense, 5′‐GATACGCAGGCTGTCAGGTT‐3′ and antisense, 5′‐GGCAGATGGTCAGGTATCGG‐3′; human thyroid hormone receptor alpha (TRα) sense, 5′‐GGAGAAGGGTGACGTTGGAA‐3′ and antisense, 5′‐TTTCATCCTTGTGGGGGTTCA‐3′; human thyroid hormone receptor beta (TRβ) sense, 5′‐GCGATTTCCTTCTGGTTGGC‐3′ and antisense, 5′‐AGTGCGGTTTCCTTATGGCT‐3′; human tryptophan hydroxylase 1 (TPH1) sense, 5′‐TCTACCCAACCCATGCTTGC‐3′ and antisense, 5′‐AAGTAACCAGCCACAGGACG‐3′; human tryptophan hydroxylase 2 (TPH2) sense, 5′‐TTGGGGTGTTGTATTCCGGG‐3′ and antisense, 5′‐CCGTGAAGCCAGACCTTTCT‐3′; human Hydroxyindole‐O‐methyltransferase (HIOMT) sense, 5′‐ACGGCTGGATTGGAGACAAG‐3′ and antisense, 5′‐CTCGGCGAGAAGGTCAAACA‐3′; human melatonin receptor 1a (MTNR1A) sense, 5′‐CACCATCGTGGTGGACATCCT‐3′ and antisense, 5′‐GCACCAACGGGTACGGATA‐3′; human melatonin receptor 1b (MTNR1B) sense, 5′‐GCTGCCCAACTTCTTTGTGG‐3′ and antisense, 5′‐GACACGACAGCGATAGGGAG‐3′; human cytochrome P450 family 27 subfamily A member 1 (CYP27A1) sense, 5′‐CCTTCGTCAGATCCATCGGG‐3′ and antisense, 5′‐GGGCCTCCATATCTTCGAGC‐3′; human cytochrome P450 family 27 subfamily B member 1 (CYP27B1) sense, 5′‐CCTGACCCACTTCCTGTTCC‐3′ and antisense, 5′‐CTGAGTGGAGTGCTGTCTGG‐3′; human cytochrome P450 family 24 subfamily A member 1 (CYP24A1) sense, 5′‐TGCCAGCGATAATACGCCTC‐3′ and antisense, 5′‐TCCCAGGCCATTCTAAGCAC‐3′; human vitamin D receptor (VDR) sense, 5′‐CGCCCACCATAAGACCTACG‐3′ and antisense, 5′‐GGGAGTGTGTCTGGAGTTGG‐3′; human nuclear receptor subfamily 3, group C, member 1 (NR3C1) sense, 5′‐GAGGGAAGGAAACTCCAGCC‐3′ and antisense, 5′‐TCAGCTAACATCTCGGGGAA‐3′; human nuclear receptor subfamily 3, group C, member 2 (NR3C2) sense, 5′‐TGCAAAAGAACCCTCGGTCA‐3′ and antisense, 5′‐GCGTGGAGAGCAGATTTTCG‐3′; human G protein‐coupled oestrogen receptor 1 (GPER1) sense, 5′‐GGGACAACTGCGGTGATGAT‐3′ and antisense, 5′‐GGATCCGCACATGACAGGTT‐3′; human Oestrogen receptor alpha (ERα) sense, 5′‐AACAGGCTCGAAAGGTCCAT‐3′ and antisense, 5′‐CAGTCCCGGAGAATGTGAAGA‐3′; human Oestrogen receptor beta (ERβ) sense, 5′‐CGTGACCGATGCTTTGGTTT‐3′ and antisense, 5′‐AGCAGATGTTCCATGCCCTT‐3′; human Microphthalmia‐associated Transposition factor (MITF) sense, 5′‐CCGGGCTCTGTTCTCACTT‐3′ and antisense, 5′‐CATGAAACTCCTCCCCGACT‐3′; human Dopachrome Tautomerase (DCT) sense, 5′‐CCCATTTTTGTGGTTCTTCATTCC‐3′ and antisense, 5′‐CGATTGTGACCAATAGGGGC‐3′; human Tyrosinase (TYR) sense, 5′‐TGACTCCAATTAGCCAGTTCCT‐3′ and antisense, 5′‐GACAGCATTCCTTCTCCATCAG‐3′; human Tyrosinase‐related protein (TYRP1) sense, 5′‐CTCAATGGCGAGTGGTCTGT‐3′ and antisense, 5′‐TTCCAAGCACTGAGCGACAT‐3′; human Premelanosome Protein 17 (Pmel17) sense, 5′‐CTATGTGCCTCTTGCTCATTCC‐3′ and antisense, 5′‐TGCTTGTTCCCTCCATCCA‐3′; human Stem cell factor receptor (c‐Kit) sense, 5′‐GCACAATGGCACGGTTGAAT‐3′ and antisense, 5′‐GGTGTGGGGATGGATTTGCT‐3′; human Endothelin receptor type B (EDNRB) sense, 5′‐CTAGGCTCTGAAACTGCGGC‐3′ and antisense, 5′‐GGCGTCATTATCTCTGCGGT‐3′; and human glyceraldehyde 3‐phosphate dehydrogenase (GAPDH) sense, 5′‐GACAGTCAGCCGCATCTTCT‐3′ and antisense, 5′‐GCGCCCAATACGACCAAATC‐3′. Experiments were independently performed at least three times using separately cultured cells, with RNA extracted from each independent culture. Data are presented as mean ± SD. Gene expression levels were normalized to GAPDH as an internal control.

### 2.6 Western Blot Analysis

For protein sample preparation, cell pellets were extracted as described previously [34], and 5 μg of the extracted protein was utilised for Western blot analysis. Primary antibodies were employed at the following dilutions: anti‐BMAL1 (#14020; CST, Danvers, MA, USA) at 1:500, anti‐CLOCK (#5157S; CST) at 1:500, anti‐MITF (sc56725, Santa Cruz Biotechnology, Texas, USA) at 1:500, anti‐Pmel17 (sc377325, Santa Cruz Biotechnology) at 1:500, anti‐TYRP1 (sc58438, Santa Cruz Biotechnology) at 1:500, anti‐Melan‐A (ab51061, Abcam, Cambridge, UK), anti‐CYP27A1 (ab126785; Abcam) at 1:500, and anti‐GAPDH (#2118; CST) at 1:1000. The anti‐GAPDH antibody was employed as a loading control.

### 2.7 Immunofluorescence Staining of Cells

HEMn‐MPs were seeded on six‐well plates and allowed to grow to confluence. After 24 h of incubation (or 48 h following siRNA transfection), the cells underwent a thorough washing with ice‐cold PBS and were then fixed with 4% paraformaldehyde for 5 min at room temperature. Fixed cells were then incubated with primary antibodies against BMAL1 (#14020; CST) or CLOCK (#5157S; CST) at a dilution of 1:500. Actin filaments were labelled using Alexa Fluor 555 Phalloidin (A34055, Invitrogen; 1:100 dilution), and nuclei were counterstained with Hoechst 33342 (Invitrogen; 1:500 dilution). Fluorescence images were acquired using a Biozero BZ‐8100 confocal microscope (Keyence Corporation, Osaka, Japan).

### 2.8 3‐[4‐Dimethylthiazol‐2‐Yl]‐2,5‐Diphenyltetrazolium Bromide (MTT) Assay

HEMn‐MP cells (2.5 × 10^4^ cells/well) were cultured in 96‐well flat‐bottom tissue culture plates. Following the experimental treatments, cells underwent three washes with cold phosphate‐buffered saline (PBS). Subsequently, cell viability was assessed using the Cell Count Reagent SF colorimetric assay (Nacalai Tesque, Kyoto, Japan). Briefly, 10 μL of Cell Count Reagent SF was added to each well, and the cells were incubated at 37°C for 2 h. Cell viability was quantified colorimetrically by measuring OD450 values using a microplate reader (Model 550; Bio‐Rad Laboratories, Hercules, CA, USA). The percentage of viable cells was calculated as follows: percentage viable cells = (*T*/*C*) × 100, where *T* and *C* represent the mean OD450 values of the treated and control groups, respectively.

### 2.9 Melanin Content Assay

To determine melanin content, cells were dissolved in 200 μL of 1 N NaOH for 30 min at 100°C to solubilize the melanin, which was then quantified in cell suspensions by recording the absorbance at 405 nm as described previously [34]. Melanin content was calculated and corrected based on cell number.

### 2.10 Identification of E‐Box Motifs in the CYP27A1 Promoter

E‐box motif analysis was performed using the JASPAR database and MEME Suite (version 5.5.0). Position frequency matrices (PFMs) for BMAL1 (ARNTL) and CLOCK transcription factors were obtained from the JASPAR CORE collection (2026 release) and converted into MEME‐compatible format. The promoter sequence of CYP27A1, defined as approximately 2 kb upstream of the transcription start site (TSS), was retrieved and used as input for motif scanning. Identification of potential transcription factor binding sites was carried out using the Find Individual Motif Occurrences (FIMO) tool with default parameters. A significance threshold of *p* < 1 × 10^−4^ was applied to identify candidate binding sites. Detected motif occurrences were mapped relative to the TSS. Canonical E‐box motifs (CANNTG), with particular attention to the high‐affinity CACGTG sequence, were manually inspected and validated. Identified binding sites were subsequently visualized and annotated for downstream analysis.

### 2.11 Fluorescent Immunohistochemical Staining

Skin tissue samples were fixed in a 10% formaldehyde solution and subsequently embedded in paraffin. From these samples, 3‐μm sections were prepared for fluorescent immunohistochemical staining. These sections underwent an overnight incubation at 4°C with primary antibodies specific to CYP27A1 (ab126785, 1:100 dilution; Abcam) and Melan‐A (M7196, 1:50 dilution; DAKO, Glostrup Kommune, Denmark). Subsequently, sections were treated with a secondary antibody (anti‐rabbit IgG Alexa Fluor 488; anti‐mouse IgG Alexa Fluor 555; Invitrogen, Thermo Fisher Scientific, Loughborough, UK). Additionally, sections were counterstained with Hoechst 33342 at a ratio of 1:500 (Invitrogen). The stained sections were visualized using either a light microscope or a Biozero 8100 confocal microscope (Keyence Co., Osaka, Japan).

### 2.12 Statistical Analysis

Each experiment was replicated no less than three times. The data are displayed as mean ± standard deviation (SD). For the evaluation of interactions between variables, a two‐way analysis of variance (ANOVA) was performed. To compare differences between two distinct groups, an unpaired Student's *t*‐test was applied using Microsoft Excel (Microsoft Corp., Redmond, WA, USA). Significance levels were set at *p* < 0.05, indicating statistical significance.

## 3 Results

### 3.1 Expression of Core Circadian Genes BMAL1 and CLOCK in Various Human Skin Cell Types

To determinate the peripheral expression of core circadian clock genes within human skin, we undertook a systematic investigation into the expression profiles of the key circadian clock components BMAL1 and CLOCK across 14 distinct human skin cell types (Figure 1h).

Quantitative real‐time PCR revealed that both BMAL1 and CLOCK were expressed in all examined cell types, albeit at different levels (Figure 1a,b). Among them, melanocytes exhibited markedly higher mRNA expression compared with fibroblasts or keratinocytes. Western blot analysis further confirmed the protein expression of BMAL1 and CLOCK across all skin cell types, with signal intensities largely consistent with the mRNA data (Figure 1c–e). Immunofluorescence staining additionally demonstrated that BMAL1 and CLOCK were clearly detectable in melanocytes, showing predominant nuclear localization, consistent with their role as transcriptional regulators (Figure 1f,g).

To further examine whether BMAL1 and CLOCK exhibit temporal expression changes in melanocytes, cultured HEMn‐MPs were synchronized with dexamethasone and analysed over a 24‐h period. Quantitative real‐time PCR analysis demonstrated temporal oscillatory changes in both BMAL1 and CLOCK expression following synchronization (Figure 1i,j). Collectively, these results indicate that the core clock components BMAL1 and CLOCK are expressed in multiple human skin cell types, with particularly high expression in melanocytes, and exhibit time‐dependent oscillatory expression patterns following synchronization, suggesting a potentially important role of the circadian machinery in pigment cell physiology.

### 3.2 Efficient Knockdown of BMAL1 and CLOCK in Human Melanocytes Reduces Melanin Synthesis

**FIGURE 2:**
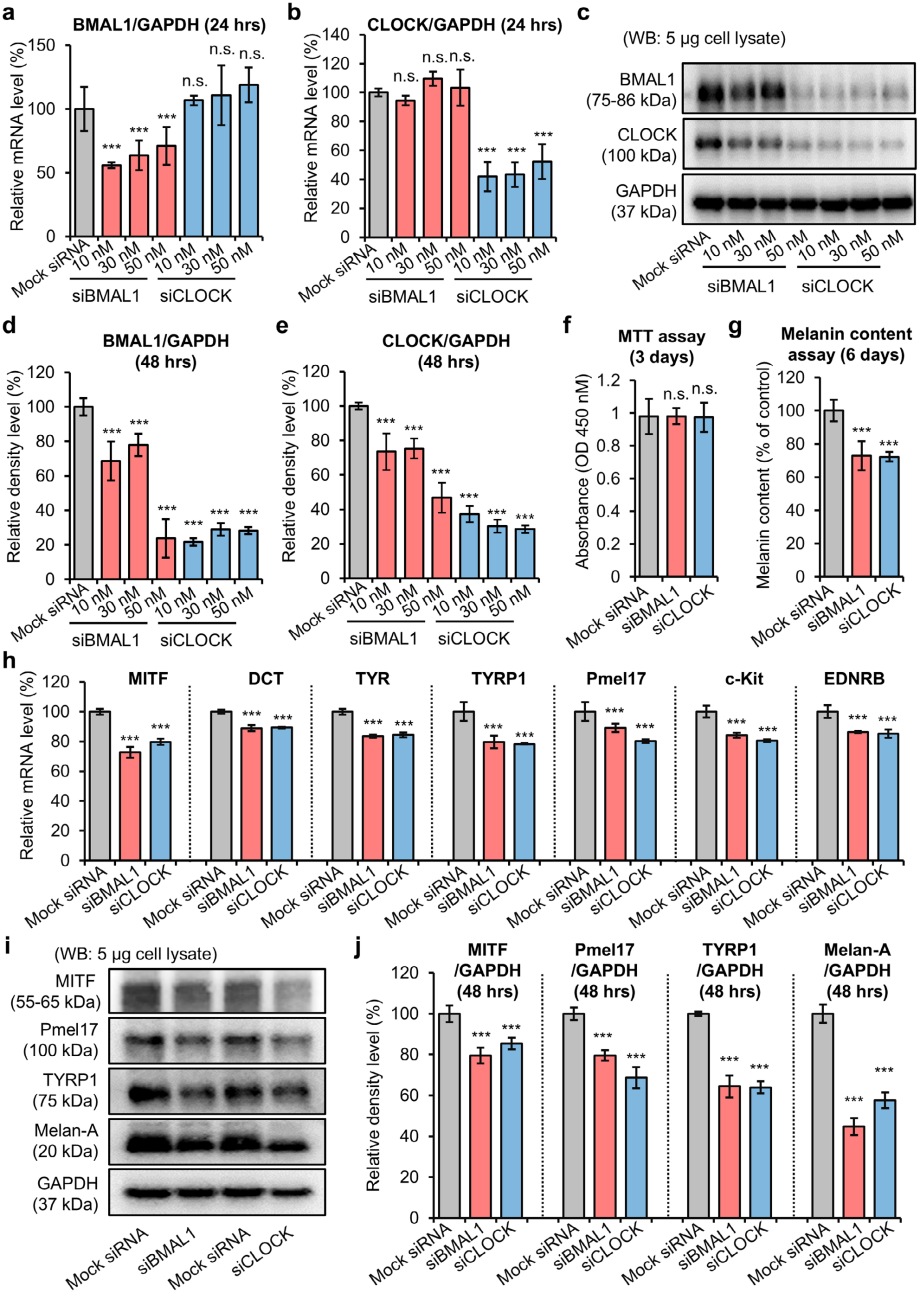
siRNA‐mediated knockdown of BMAL1 and CLOCK reduces melanogenesis in human epidermal melanocytes (HEMn‐MP). Quantitative real‐time PCR analysis showing efficient suppression of BMAL1 (a) and CLOCK (b) mRNA expression 24 h after transfection with siBMAL1 or siCLOCK (10, 30, or 50 nM). Gene expression levels were normalized to GAPDH. Western blot analysis of BMAL1 and CLOCK protein expression in HEMn‐MP cells 48 h after siRNA transfection. GAPDH served as the loading control (c). Densitometric quantification of BMAL1 (d) and CLOCK (e) protein expression shown in (c). Band densities were normalized to GAPDH and expressed relative to the Mock siRNA control. Cell viability measured by MTT assay 3 days after transfection, showing no significant cytotoxicity induced by BMAL1 or CLOCK knockdown (f). Intracellular melanin content quantified 6 days after siRNA treatment, demonstrating reduced melanogenesis in BMAL1‐ and CLOCK‐silenced melanocytes (g). Quantitative real‐time PCR analysis of key melanogenesis‐related genes (MITF, DCT, TYR, TYRP1, Pmel17, c‐Kit, and EDNRB) 24 h after siRNA transfection. Gene expression levels were normalized to GAPDH and expressed relative to the corresponding Mock siRNA control for each individual gene (set to 100%) (h). Western blot validation of selected melanogenic proteins (MITF, Pmel17, TYRP1, Melan‐A) showing decreased protein expression 48 h after siRNA transfection, with GAPDH as the loading control (i). Densitometric quantification of these protein levels is shown in (j). Band densities were normalized to GAPDH and expressed relative to the Mock siRNA control. Data in a, b, d, e, f, g, h and j are shown as mean ± SD. ***p < 0.01; n.s., no significant difference, as determined by Student's *t*‐test.

To elucidate the functional role of circadian clock genes in melanocytes, siRNA‐mediated knockdown of BMAL1 and CLOCK was performed in cultured human epidermal melanocytes (HEMn‐MPs). Quantitative real‐time PCR confirmed significant reductions in both BMAL1 and CLOCK transcript levels after siRNA transfection (Figure 2a,b). Specifically, BMAL1 mRNA levels were reduced to approximately 55%, 63%, and 70% of control levels following transfection with 10, 30, and 50 nM siBMAL1, respectively, whereas CLOCK mRNA levels were reduced to approximately 41%, 43%, and 52% of control levels following transfection with 10, 30, and 50 nM siCLOCK, respectively. Western blot analysis further showed marked decreases in BMAL1 and CLOCK protein expression in the knockdown groups (Figure 2c). Densitometric quantification revealed that BMAL1 protein levels were reduced to approximately 68%, 77%, and 23% of control levels, while CLOCK protein levels were reduced to approximately 37%, 30%, and 28% of control levels following treatment with 10, 30, and 50 nM of the corresponding siRNAs, respectively (Figure 2d,e).

To determine whether silencing these clock genes affects melanocyte viability or proliferation, an MTT assay was conducted (Figure 2f). No significant differences were observed among the mock‐, siBMAL1‐, and siCLOCK‐transfected groups, indicating that the knockdown did not induce cytotoxicity or impair proliferation. Given the primary role of melanocytes in pigment production, we next examined the functional consequences of BMAL1 and CLOCK depletion on melanogenesis. Measurement of intracellular melanin content revealed a significant decrease in both siBMAL1‐ and siCLOCK‐treated cells compared with mock‐transfected controls, with melanin levels decreased to approximately 72% of control levels in both groups (Figure 2g).

To further examine the mechanisms underlying the reduced pigmentation, we analysed the expression of key melanogenic genes. Quantitative real‐time PCR revealed that the mRNA levels of major melanogenesis‐associated genes, including MITF, DCT, TYR, TYRP1, Pmel17, c‐Kit, and endothelin receptor B (EDNRB), were consistently decreased following BMAL1 or CLOCK knockdown (Figure 2h). Specifically, transcript levels were reduced to approximately 72% and 79% of control levels for MITF, 88% and 89% for DCT, 83% and 84% for TYR, 79% and 78% for TYRP1, 89% and 80% for Pmel17, 84% and 80% for c‐Kit, and 86% and 85% for EDNRB in the siBMAL1 and siCLOCK groups, respectively. Consistent with these transcriptional changes, western blot analysis demonstrated clear reductions in the protein levels of MITF, Pmel17, TYRP1, and Melan‐A in both knockdown groups (Figure 2i). Protein levels were reduced to approximately 79% and 85% of control levels for MITF, 79% and 68% for Pmel17, 64% and 63% for TYRP1, and 44% and 57% for Melan‐A in the siBMAL1 and siCLOCK groups, respectively (Figure 2j). These coordinated reductions indicate that BMAL1 or CLOCK suppression broadly attenuates the melanogenic pathway. Together, these findings demonstrate that BMAL1 and CLOCK were effectively silenced in human melanocytes, and their downregulation led to reduced melanin synthesis through suppression of multiple melanogenic genes, without affecting cell viability. These results indicate that both BMAL1 and CLOCK act as positive regulators of melanogenic activity in human melanocytes.

### 3.3 Knockdown of BMAL1 and CLOCK Selectively Suppresses CYP27A1 and Downstream Melanogenic Pathways in Human Epidermal Melanocytes

**FIGURE 3:**
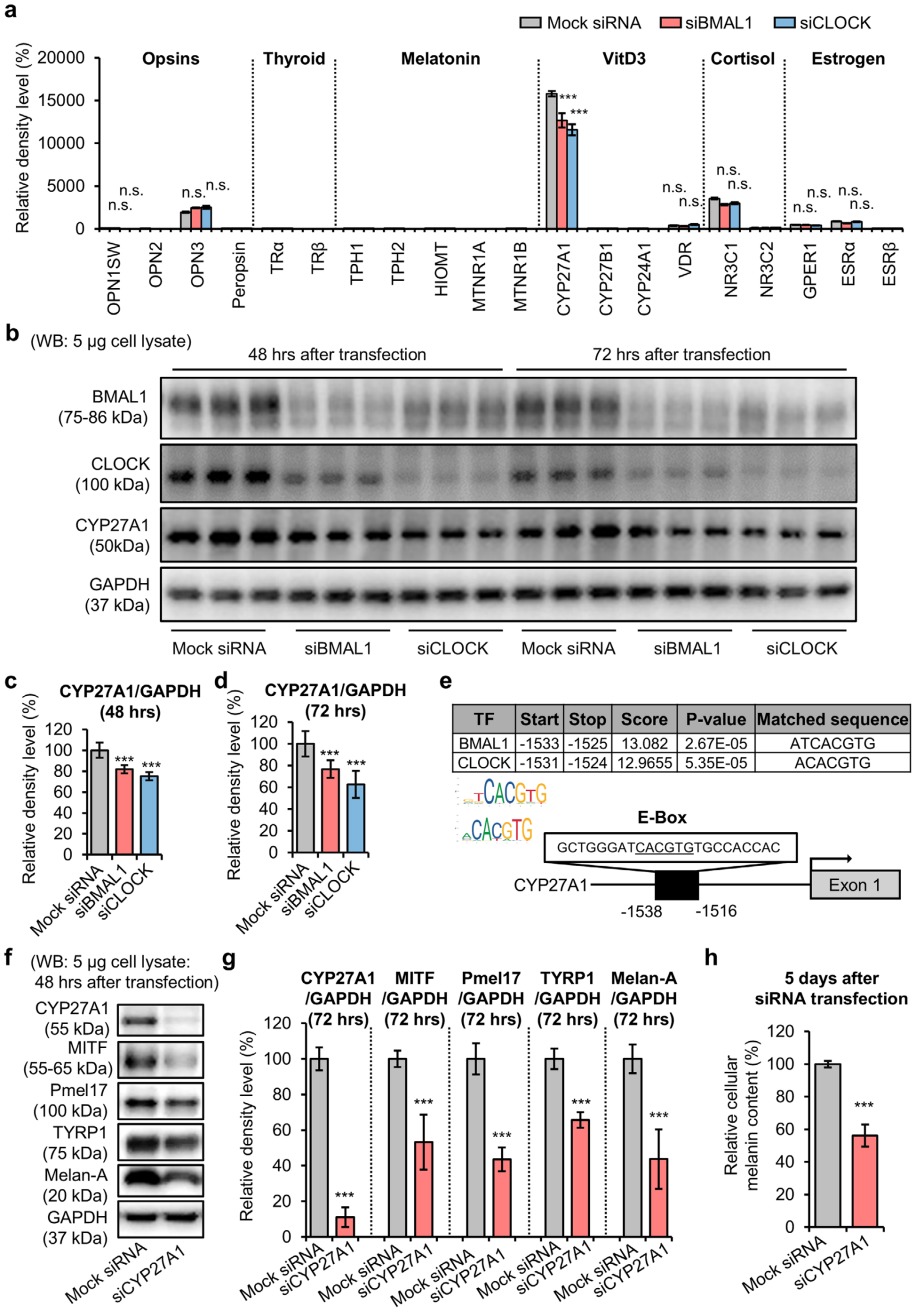
Knockdown of BMAL1 and CLOCK selectively suppresses CYP27A1 and downstream melanogenesis‐related protein expression in human epidermal melanocytes. Quantitative real‐time PCR analysis of hormone‐related genes across six functional categories (opsins, thyroid signalling, melatonin pathway, vitamin D metabolism, cortisol signalling, and oestrogen signalling) 24 h after transfection with siBMAL1 or siCLOCK. Gene expression levels were normalized to GAPDH and expressed relative to OPN1SW (set to 100%) (a). Genes with Ct values > 30 were considered to have low‐confidence expression levels and are shown without statistical annotation. Statistical analysis was performed only for genes with reliably detectable expression levels. Among all targets tested, CYP27A1 showed the most pronounced reduction following BMAL1 or CLOCK knockdown. (a) Western blot analysis of BMAL1, CLOCK, and CYP27A1 protein levels in HEMn‐MP melanocytes at 48 and 72 h after siRNA transfection. GAPDH served as the loading control (b). Densitometric quantification of CYP27A1 protein normalized to GAPDH at 48 h (c) and 72 h (d) post‐transfection, confirming a sustained reduction in CYP27A1 protein expression in BMAL1‐ and CLOCK‐silenced cells. (e) Bioinformatic analysis of the CYP27A1 promoter identifying a putative BMAL1/CLOCK binding site. An E‐box motif (CACGTG) was detected at positions −1530 to −1525 relative to the transcription start site. The table summarizes predicted transcription factor binding sites with corresponding scores and *p*‐values. The schematic diagram shows the location of the E‐box motif upstream of exon 1 of CYP27A1. (f) Western blot analysis of CYP27A1, MITF, TYRP1, Melan‐A, and Pmel17 protein expression following CYP27A1 knockdown in HEMn‐MP melanocytes. GAPDH served as the loading control. (g) Densitometric quantification of the indicated proteins normalized to GAPDH, demonstrating reduced expression of melanogenesis‐related proteins following CYP27A1 silencing. (h) Quantification of intracellular melanin content in HEMn‐MP melanocytes 5 days after transfection with 30 nM siCYP27A1. CYP27A1 knockdown significantly reduced melanin production compared with the Mock siRNA control. Data in a, c, d, g, and h are shown as mean ± SD. ***p < 0.01; n.s., no significant difference, as determined by Student's *t*‐test.

To explore whether the circadian clock influences local endocrine systems in melanocytes, we analysed the expression of genes belonging to six major hormonal pathways relevant to skin physiology, including opsins, thyroid hormone signalling, melatonin synthesis, vitamin D3 metabolism, cortisol signalling, and oestrogen signalling (Figure 3a). Quantitative real‐time PCR analysis revealed that the vast majority of genes across these pathways were unaffected by BMAL1 or CLOCK knockdown. However, a pronounced and selective decrease was observed in CYP27A1, a mitochondrial enzyme that catalyses the initial hydroxylation of vitamin D3. CYP27A1 mRNA levels were reduced to approximately 80% and 73% of control levels in the siBMAL1‐ and siCLOCK‐treated groups, respectively. CYP27A1 converts vitamin D3 (cholecalciferol) to 25‐hydroxyvitamin D3 [25(OH)D3], the principal circulating precursor of hormonally active calcitriol. Thus, the selective decrease in CYP27A1 suggests that circadian clock disruption may impair melanocyte‐intrinsic vitamin D metabolic capacity.

To confirm the transcriptional results, CYP27A1 protein levels were examined by Western blotting at 48 and 72 h after siRNA treatment (Figure 3b). Consistent with the mRNA data, CYP27A1 protein expression was significantly reduced in both siBMAL1‐ and siCLOCK‐treated melanocytes at both time points (Figure 3c,d). At 48 h, CYP27A1 protein levels were reduced to approximately 81% and 75% of control levels in the siBMAL1 and siCLOCK groups, respectively; whereas at 72 h they were further reduced to approximately 76% and 62% of control levels. BMAL1 and CLOCK knockdown efficiency was confirmed by the corresponding depletion of each target protein.

To explore a potential regulatory mechanism, bioinformatic analysis of the CYP27A1 promoter region (~2 kb upstream of the transcription start site) identified a canonical E‐box motif (CACGTG), a known high‐affinity binding site for the BMAL1/CLOCK heterodimer (Figure 3e). This motif was located approximately −1530 to −1525 bp upstream of the transcription start site, suggesting a potential regulatory role of circadian transcription factors in CYP27A1 expression. To further investigate the functional significance of CYP27A1 in melanocytes, CYP27A1 was silenced using siRNA in HEMn‐MP cells (Figure 3f). Knockdown of CYP27A1 resulted in reduced expression of multiple melanogenesis‐related proteins, including MITF, TYRP1, Melan‐A, and Pmel17, compared with the Mock siRNA control (Figure 3f,g). In addition, intracellular melanin content was significantly decreased 5 days after transfection with 30 nM siCYP27A1 (Figure 3h), indicating that CYP27A1 contributes to the maintenance of melanogenic activity in melanocytes. Collectively, these results demonstrate that CYP27A1 is selectively regulated by BMAL1 and CLOCK among multiple cutaneous hormonal pathways and may function as a downstream mediator linking circadian regulation to melanogenesis in human epidermal melanocytes.

### 3.4 Reduced Expression of BMAL1, CLOCK, and CYP27A1 in Melanocytes Within Vitiligo Lesions

**FIGURE 4:**
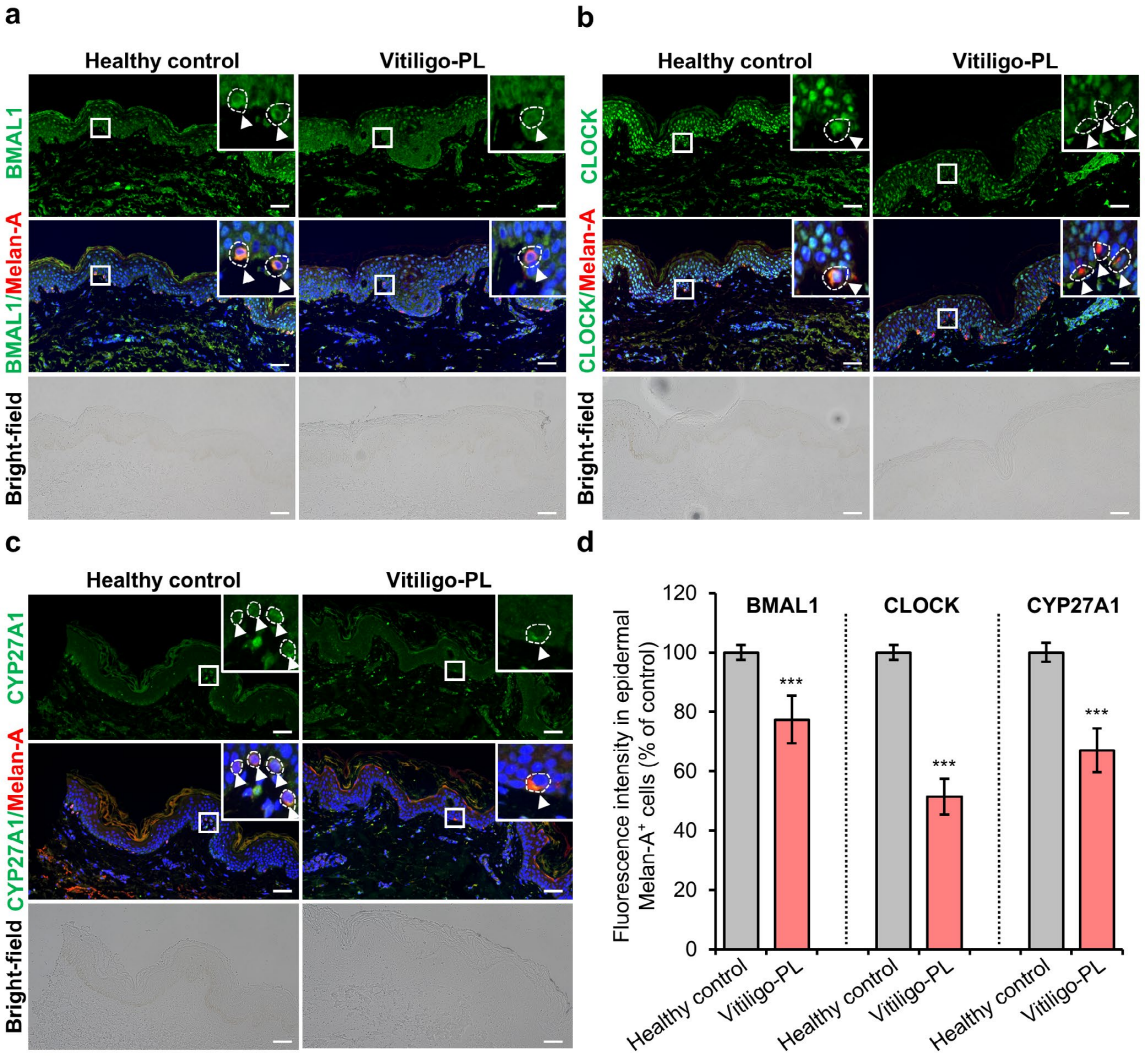
Reduced expression of BMAL1, CLOCK, and CYP27A1 in melanocytes of vitiligo lesional skin. Representative immunofluorescence staining of BMAL1 (a), CLOCK (b), and CYP27A1 (c) in healthy control and vitiligo lesional epidermis. Target proteins are shown in green, Melan‐A‐positive melanocytes in red, and nuclei are counterstained with DAPI (blue). Merged images demonstrate protein expression within Melan‐A‐positive melanocytes. Insets highlight representative melanocytes in the epidermis. Corresponding bright‐field images are shown below. Scale bars: 100 μm. (d) Quantification of BMAL1, CLOCK, and CYP27A1 fluorescence intensity in Melan‐A‐positive melanocytes. Fluorescence intensities were normalized to the healthy control group, which was set to 100%. Data are presented as mean ± SD. ***p < 0.01.

To determine whether circadian clock components and the vitamin D3 metabolic pathway are altered in vitiligo, we analysed BMAL1, CLOCK, and CYP27A1 protein expression in vitiligo patients and control epidermal tissues by immunofluorescence staining (Figure 4). In healthy skin, BMAL1, CLOCK, and CYP27A1 were strongly expressed in Melan‐A‐positive melanocytes distributed along the basal epidermal layer (Figure 4a–c). In contrast, vitiligo lesional skin showed a marked reduction in Melan‐A‐positive melanocytes, consistent with melanocyte loss in vitiligo lesions. Moreover, the remaining melanocytes within vitiligo lesions exhibited substantially decreased BMAL1, CLOCK, and CYP27A1 fluorescence signals compared with melanocytes in healthy control skin. Quantitative fluorescence analysis further confirmed significant reductions in BMAL1, CLOCK, and CYP27A1 expression in Melan‐A‐positive melanocytes within vitiligo lesions (Figure 4d). Relative fluorescence intensities were reduced to approximately 77%, 51%, and 67% of healthy control levels for BMAL1, CLOCK, and CYP27A1, respectively. These findings demonstrate that both core circadian clock components and CYP27A1 are diminished in melanocytes within vitiligo lesions, supporting the in vitro findings that BMAL1/CLOCK signalling may regulate vitamin D3 metabolism and melanogenic activity in melanocytes.

## 4 Discussion

Collectively, our integrated transcriptomic, proteomic, and spatial analyses demonstrate that the core circadian regulators BMAL1 and CLOCK are predominantly expressed in epidermal melanocytes among the major human skin cell types examined. Synchronization experiments further revealed temporal oscillatory changes in BMAL1 and CLOCK expression following dexamethasone treatment, supporting the presence of functional circadian machinery in melanocytes. Together with their predominant nuclear localization, these findings suggest that melanocytes are not merely passive recipients of systemic circadian cues but may possess clock‐regulated transcriptional programs that contribute to melanocyte physiology.

In addition to melanin reduction itself, BMAL1/CLOCK depletion broadly suppressed the melanogenic transcriptional program, including MITF, TYRP1, Pmel17, and Melan‐A expression. These coordinated changes indicate that circadian regulators may function upstream of core melanocyte differentiation and pigmentation pathways. Consistent with this, intracellular melanin content was markedly reduced following BMAL1 or CLOCK knockdown, supporting an important role for circadian signalling in maintaining melanocyte functional identity and pigment production.

To further investigate the mechanisms linking circadian signalling to melanocyte function, we screened multiple skin‐related hormonal pathways in BMAL1‐ and CLOCK‐silenced melanocytes. Interestingly, among the pathways examined, CYP27A1 emerged as the most selectively and consistently regulated target. CYP27A1 is a mitochondrial enzyme that catalyses the hydroxylation of vitamin D_3_ to 25‐hydroxyvitamin D_3_ [25(OH)D_3_], the major circulating precursor of active vitamin D metabolites [35, 36]. In addition to its classical endocrine functions, locally produced vitamin D metabolites have been implicated in cutaneous immune regulation, photoprotection, and melanocyte biology [37].

Our data demonstrated that BMAL1/CLOCK knockdown significantly reduced CYP27A1 expression at both the mRNA and protein levels. Furthermore, bioinformatic analysis identified a canonical E‐box motif within the CYP27A1 promoter region, suggesting a potential transcriptional link between circadian clock components and CYP27A1 expression. Although additional mechanistic studies such as chromatin immunoprecipitation or promoter reporter assays will be necessary to confirm direct transcriptional regulation, these findings support a previously unrecognized association between circadian signalling and melanocyte‐associated vitamin D metabolism.

Importantly, direct silencing of CYP27A1 recapitulated several effects observed following BMAL1/CLOCK depletion, including reduced expression of melanogenesis‐related proteins and decreased melanin production. These findings suggest that CYP27A1 may function as a downstream effector linking circadian regulation to melanogenic activity in melanocytes. Because vitamin D_3_ has previously been reported to promote melanocyte dendricity, tyrosinase activity, and pigmentation [38], our findings raise the possibility that circadian disruption may impair melanogenesis, at least in part, through suppression of CYP27A1‐dependent vitamin D metabolism.

Interestingly, our observations in epidermal melanocytes differ from previous reports in human hair follicle organ culture models, in which silencing BMAL1 or PER1 enhanced pigmentation‐related pathways [33]. Importantly, the previous study investigated intact hair follicle units rather than isolated follicular melanocytes alone, suggesting that the divergent outcomes may reflect not only intrinsic differences between epidermal and follicular melanocytes, but also distinct microenvironmental and niche‐dependent regulatory mechanisms. Hair follicles contain complex epithelial–mesenchymal and melanocyte–keratinocyte interactions, as well as hair cycle‐associated signalling networks, which are absent in isolated epidermal melanocyte systems. In contrast, epidermal melanocytes primarily function in constitutive pigmentation and UV‐associated photoprotection within the epidermal melanin unit. These findings collectively suggest that circadian regulation of melanocyte biology may vary according to tissue architecture, surrounding cellular interactions, and physiological function.

Importantly, BMAL1, CLOCK, and CYP27A1 expression were all reduced in Melan‐A‐positive melanocytes within vitiligo lesions, extending our in vitro findings to human disease tissue. These observations suggest that dysregulation of circadian‐associated melanogenic and vitamin D metabolic pathways may contribute to melanocyte dysfunction in vitiligo. In vitiligo lesions, impaired local vitamin D metabolism may not only affect pigmentation but also alter the immunophysiological microenvironment of the skin.

Taken together, our findings underscore that the core circadian clock exerts lineage‐specific, microenvironment‐dependent control over melanocyte physiology by regulating both melanogenesis and local vitamin D metabolism. The identification of a BMAL1/CLOCK–CYP27A1 axis provides new insight into the relationship between circadian regulation and pigment cell biology and suggests a potential link between circadian dysregulation and pigmentary disorders such as vitiligo. Future studies investigating the chromatin landscape, temporal transcriptional dynamics, and signalling pathways associated with circadian regulation in melanocytes will be important for further defining these mechanisms and for exploring chronobiology‐based therapeutic strategies for pigmentary diseases.

## Author Contributions

A.Z. and L.Y. conceived and designed the study. A.Z. performed the experiments and analysed the data. A.Z. drafted the manuscript. A.Z., L.Y., S.L., F.Y., D.T. and I.K. contributed to data interpretation and critically revised the manuscript. All authors approved the final version of the manuscript.

## Funding

The authors have nothing to report.

## Ethics Statement

The specimen collection and overall study design were approved by the Medical Ethics Committee of Osaka Metropolitan University (no. 4152).

## Consent

All authors have read and approved the final manuscript and agree to its submission to the journal.

## Conflicts of Interest

The authors declare no conflicts of interest.

## Data Availability

The data that support the findings of this study are available within the article.

## References

[1] M. H. Vitaterna , J. S. Takahashi , and F. W. Turek , “Overview of Circadian Rhythms,” Alcohol Research & Health 25, no. 2 (2001): 85–93. 11584554 PMC6707128

[2] S. L. Harmer , S. Panda , and S. A. Kay , “Molecular Bases of Circadian Rhythms,” Annual Review of Cell and Developmental Biology 17 (2001): 215–253. 10.1146/annurev.cellbio.17.1.215 11687489

[3] A. Patke , M. W. Young , and S. Axelrod , “Molecular Mechanisms and Physiological Importance of Circadian Rhythms,” Nature Reviews. Molecular Cell Biology 21, no. 2 (2020): 67–84. 31768006 10.1038/s41580-019-0179-2

[4] J. Richards and M. L. Gumz , “Advances in Understanding the Peripheral Circadian Clocks,” FASEB Journal 26, no. 9 (2012): 3602–3613. 22661008 10.1096/fj.12-203554 PMC3425819

[5] J. A. Mohawk , C. B. Green , and J. S. Takahashi , “Central and Peripheral Circadian Clocks in Mammals,” Annual Review of Neuroscience 35 (2012): 445–462. 10.1146/annurev-neuro-060909-153128 PMC3710582 22483041

[6] F. Sporl , K. Schellenberg , T. Blatt , et al., “A Circadian Clock in HaCaT Keratinocytes,” Journal of Investigative Dermatology 131, no. 2 (2011): 338–348. 20962856 10.1038/jid.2010.315

[7] C. Sandu , M. Dumas , A. Malan , et al., “Human Skin Keratinocytes, Melanocytes, and Fibroblasts Contain Distinct Circadian Clock Machineries,” Cellular and Molecular Life Sciences 69, no. 19 (2012): 3329–3339. 22627494 10.1007/s00018-012-1026-1 PMC11114759

[8] S. B. Zanello , D. M. Jackson , and M. F. Holick , “Expression of the Circadian Clock Genes Clock and period1 in Human Skin,” Journal of Investigative Dermatology 115, no. 4 (2000): 757–760. 10998156 10.1046/j.1523-1747.2000.00121.x

[9] K. Yagita , F. Tamanini , G. T. van der Horst , and H. Okamura , “Molecular Mechanisms of the Biological Clock in Cultured Fibroblasts,” Science 292, no. 5515 (2001): 278–281. 11303101 10.1126/science.1059542

[10] E. Nagoshi , C. Saini , C. Bauer , T. Laroche , F. Naef , and U. Schibler , “Circadian Gene Expression in Individual Fibroblasts: Cell‐Autonomous and Self‐Sustained Oscillators Pass Time to Daughter Cells,” Cell 119, no. 5 (2004): 693–705. 15550250 10.1016/j.cell.2004.11.015

[11] M. V. Plikus , E. N. Van Spyk , K. Pham , et al., “The Circadian Clock in Skin: Implications for Adult Stem Cells, Tissue Regeneration, Cancer, Aging, and Immunity,” Journal of Biological Rhythms 30, no. 3 (2015): 163–182. 25589491 10.1177/0748730414563537 PMC4441597

[12] G. Yosipovitch , G. L. Xiong , E. Haus , L. Sackett‐Lundeen , I. Ashkenazi , and H. I. Maibach , “Time‐Dependent Variations of the Skin Barrier Function in Humans: Transepidermal Water Loss, Stratum Corneum Hydration, Skin Surface pH, and Skin Temperature,” Journal of Investigative Dermatology 110, no. 1 (1998): 20–23. 9424081 10.1046/j.1523-1747.1998.00069.x

[13] A. B. Lyons , L. Moy , R. Moy , and R. Tung , “Circadian Rhythm and the Skin: A Review of the Literature,” Journal of Clinical and Aesthetic Dermatology 12, no. 9 (2019): 42–45. PMC6777699 31641418

[14] M. S. Matsui , E. Pelle , K. Dong , and N. Pernodet , “Biological Rhythms in the Skin,” International Journal of Molecular Sciences 17, no. 6 (2016): 801. 27231897 10.3390/ijms17060801 PMC4926335

[15] J. Duan , E. N. Greenberg , S. S. Karri , and B. Andersen , “The Circadian Clock and Diseases of the Skin,” FEBS Letters 595, no. 19 (2021): 2413–2436. 34535902 10.1002/1873-3468.14192 PMC8515909

[16] S. Sarkar , K. I. Porter , P. P. Dakup , et al., “Circadian Clock Protein BMAL1 Regulates Melanogenesis Through MITF in Melanoma Cells,” Pigment Cell & Melanoma Research 34, no. 5 (2021): 955–965. 34160901 10.1111/pcmr.12998 PMC8429232

[17] P. Dakup and S. Gaddameedhi , “Impact of the Circadian Clock on UV‐Induced DNA Damage Response and Photocarcinogenesis,” Photochemistry and Photobiology 93, no. 1 (2017): 296–303. 27861965 10.1111/php.12662 PMC5315601

[18] V. Nikkola , M. E. Miettinen , P. Karisola , et al., “Ultraviolet B Radiation Modifies Circadian Time in Epidermal Skin and in Subcutaneous Adipose Tissue,” Photodermatology, Photoimmunology & Photomedicine 35, no. 3 (2019): 157–163. 10.1111/phpp.12440 30472764

[19] M. Bracci , V. Ciarapica , A. Copertaro , et al., “Peripheral Skin Temperature and Circadian Biological Clock in Shift Nurses After a Day Off,” International Journal of Molecular Sciences 17, no. 5 (2016): 623. 27128899 10.3390/ijms17050623 PMC4881449

[20] A. Luengas‐Martinez , R. Paus , M. Iqbal , L. Bailey , D. W. Ray , and H. S. Young , “Circadian Rhythms in Psoriasis and the Potential of Chronotherapy in Psoriasis Management,” Experimental Dermatology 31, no. 11 (2022): 1800–1809. 35851722 10.1111/exd.14649 PMC9805195

[21] S. Gelfant , A. Ozawa , D. K. Chalker , and J. G. Smith, Jr. , “Circadian Rhythms and Differences in Epidermal and in Dermal Cel Proliferation in Uninvolved and Involved Psoriatic Skin In Vivo,” Journal of Investigative Dermatology 78, no. 1 (1982): 58–62. 7054307 10.1111/1523-1747.ep12497933

[22] N. Sun , D. Dai , S. Deng , X. Cai , and P. Song , “Bioinformatics Integrative Analysis of Circadian Rhythms Effects on Atopic Dermatitis and Dendritic Cells,” Clinical, Cosmetic and Investigational Dermatology 16 (2023): 2919–2930. 37873510 10.2147/CCID.S424343 PMC10590565

[23] W. Q. Li , A. A. Qureshi , E. S. Schernhammer , and J. Han , “Rotating Night‐Shift Work and Risk of Psoriasis in US Women,” Journal of Investigative Dermatology 133, no. 2 (2013): 565–567. 22931920 10.1038/jid.2012.285 PMC3511636

[24] A. G. A. Farag , E. A. E. Badr , and A. F. Ibrahim , “Circadian Clock Gene Expression and Polymorphism in Non‐Segmental Vitiligo,” Molecular Biology Reports 51, no. 1 (2024): 142. 38236441 10.1007/s11033-023-09109-6 PMC10796645

[25] C. C. Zouboulis , “Human Skin: An Independent Peripheral Endocrine Organ,” Hormone Research 54, no. 5–6 (2000): 230–242. 11595811 10.1159/000053265

[26] C. C. Zouboulis , “The Skin as an Endocrine Organ,” Dermato‐Endocrinology 1, no. 5 (2009): 250–252. 20808511 10.4161/derm.1.5.9499 PMC2836429

[27] C. C. Zouboulis , “The Human Skin as a Hormone Target and an Endocrine Gland,” Hormones (Athens, Greece) 3, no. 1 (2004): 9–26. 16982574 10.14310/horm.2002.11109

[28] J. Reichrath , “The Skin Is a Fascinating Endocrine Organ,” Dermato‐Endocrinology 1, no. 4 (2009): 195–196. 20592790 10.4161/derm.1.4.9653 PMC2835874

[29] A. Slominski , B. Zbytek , G. Nikolakis , et al., “Steroidogenesis in the Skin: Implications for Local Immune Functions,” Journal of Steroid Biochemistry and Molecular Biology 137 (2013): 107–123. 23435015 10.1016/j.jsbmb.2013.02.006 PMC3674137

[30] D. Roosterman , T. Goerge , S. W. Schneider , N. W. Bunnett , and M. Steinhoff , “Neuronal Control of Skin Function: The Skin as a Neuroimmunoendocrine Organ,” Physiological Reviews 86, no. 4 (2006): 1309–1379. 17015491 10.1152/physrev.00026.2005

[31] M. F. Holick , J. A. MacLaughlin , M. B. Clark , et al., “Photosynthesis of Previtamin D3 in Human Skin and the Physiologic Consequences,” Science 210, no. 4466 (1980): 203–205. 6251551 10.1126/science.6251551

[32] G. E. Bertolesi , N. Debnath , N. Heshami , et al., “Interplay of Light, Melatonin, and Circadian Genes in Skin Pigmentation Regulation,” Pigment Cell & Melanoma Research 38, no. 1 (2025): e13220. 39825699 10.1111/pcmr.13220 PMC11742648

[33] J. A. Hardman , D. J. Tobin , I. S. Haslam , et al., “The Peripheral Clock Regulates Human Pigmentation,” Journal of Investigative Dermatology 135, no. 4 (2015): 1053–1064. 25310406 10.1038/jid.2014.442

[34] F. Yang , L. Yang , M. Wataya‐Kaneda , T. Yoshimura , A. Tanemura , and I. Katayama , “Uncoupling of ER/Mitochondrial Oxidative Stress in mTORC1 Hyperactivation‐Associated Skin Hypopigmentation,” Journal of Investigative Dermatology 138, no. 3 (2018): 669–678. 29080681 10.1016/j.jid.2017.10.007

[35] B. Lehmann , “The Vitamin D3 Pathway in Human Skin and Its Role for Regulation of Biological Processes,” Photochemistry and Photobiology 81, no. 6 (2005): 1246–1251. 16162035 10.1562/2005-02-02-IR-430

[36] B. Lehmann , O. Tiebel , and M. Meurer , “Expression of Vitamin D3 25‐Hydroxylase (CYP27) mRNA After Induction by Vitamin D3 or UVB Radiation in Keratinocytes of Human Skin Equivalents—A Preliminary Study,” Archives of Dermatological Research 291, no. 9 (1999): 507–510. 10541881 10.1007/s004030050445

[37] A. Piotrowska , J. Wierzbicka , and M. A. Zmijewski , “Vitamin D in the Skin Physiology and Pathology,” Acta Biochimica Polonica 63, no. 1 (2016): 17–29. 26824295 10.18388/abp.2015_1104

[38] Y. Tomita , W. Torinuki , and H. Tagami , “Stimulation of Human Melanocytes by Vitamin D3 Possibly Mediates Skin Pigmentation After Sun Exposure,” Journal of Investigative Dermatology 90, no. 6 (1988): 882–884. 2836517 10.1111/1523-1747.ep12462151

